# Identification of Furin Protease Small-Molecule Inhibitor with a 1,3-Thiazol-2-ylaminosulfonyl Scaffold

**DOI:** 10.3390/ph18020273

**Published:** 2025-02-19

**Authors:** Anja Kolarič, Vid Ravnik, Sara Štumpf Horvat, Marko Jukič, Urban Bren

**Affiliations:** 1 Laboratory of Physical Chemistry and Chemical Thermodynamics, Faculty of Chemistry and Chemical Engineering, University of Maribor, Smetanova ulica 17, SI-2000 Maribor, Slovenia; 2 Faculty of Mathematics, Natural Sciences and Information Technologies, University of Primorska, Glagoljaška ulica 8, SI-6000 Koper, Slovenia; 3 Institute of Environmental Protection and Sensors, Beloruska ulica 7, SI-2000 Maribor, Slovenia

**Keywords:** computer-assisted drug design, CADD, structure-based drug design, furin inhibitors, protease inhibitors, antivirals, water-aware molecular docking, furin assay, antiviral drug design

## Abstract

**Background:** Proteolytic cleavage of inactive pathogen proteins by furin is critical for their entry into human cells, and thus furin cleavage of the SARS-CoV-2 spike protein was identified as a prerequisite for virus binding and the subsequent infection of human cells in the recent COVID-19 pandemic. We report a water-aware structure-based protease inhibitor design study. **Methods:** Our efforts focused on the biological evaluation of small molecule inhibitors that emerged from a conserved water-aware virtual screening campaign of a library of compounds that shared structural or physicochemical properties with known furin inhibitors exhibiting newly recognized binding modes. **Results:** We identified a novel small-molecule furin protease inhibitor with a 1,3-thiazol-2-ylaminosulfonyl scaffold. Namely, the compound *N*-[4-(1,3-thiazol-2-ylaminosulfonyl)phenyl]-3-{(E)-5-[(2-methoxyphenyl)methylene]-4-oxo-2-thioxo-1,3-thiazolidin-3-yl}propionamide showed an IC_50_ value of 17.58 μM, comparable to other published inhibitors. **Conclusions:** This compound could represent a starting point for the further design and development of non-peptidic, small-molecule furin inhibitors that could assist in furin cleavage studies and coronaviral pathogenesis.

## 1. Introduction

Furin represents one of the best-characterized members of proprotein convertases that regulate numerous processes in health and disease. It is a serine protease that cleaves inactive precursor proteins at polybasic recognition motifs to produce mature and active proteins [1]. Furin substrates include various human proteins such as cytokines, hormones, growth factors, and receptors. However, furin is also associated with a variety of diseases such as cancer, and promotes the virulence and proliferation of many viruses and bacteria [1,2,3]. Furin cleaves its substrates after C-terminal arginine (Arg) residue in the preferred consensus sequence Arg-X-Arg/Lys-Arg↓(R-X-R/K-R↓). The variations in this cleavage site allow for furin to process a wide range of target substrates [1,2]. This cleavage is performed by a catalytic triad consisting of amino acid residues Asp153, His194, and Ser368 [3].

Many viral proteins require proteolytic processing by proprotein convertases. Therefore, furin has been associated with the activation of several viruses, including those from the Herpes, Corona, Flavi, Toga, Borna, Bunya, Filo, Orthomyxo, Paramyxo, Pneumo, and Retroviridae families [2]. It has also been identified as an important antiviral target in the recent COVID-19 pandemic [3,4]. SARS-CoV-2 uses its spike glycoprotein to invade host cells and is synthesized as an inactive precursor to evade host immune surveillance. Therefore, it must be activated for successful infection of host cells [5,6].

The spike protein consists of two functional subunits, S1 and S2, where the S1 subunit is critical for host cell receptor binding, while the S2 subunit harbors the membrane fusion machinery of the viral and host cell membranes. Host cell proteases activate the viral spike protein at two cleavage sites called S1/S2 and S2′ in a two-step sequential process [7,8]. The boundary between the S1/S2 subunits contains the polybasic sequence ^677^QTNSPRRAR↓S^686^ [3,9], which is recognized and cleaved by furin. This results in conformational changes of the spike protein that promote angiotensin-converting enzyme 2 (ACE2) receptor recognition [3]. Furin cleavage is followed by a subsequent cleavage at the S2′ site by the transmembrane protease serine 2 (TMPRSS2), which leads to a fusion of viral and host cell membranes, resulting in virus entry [3].

**Figure 1:**
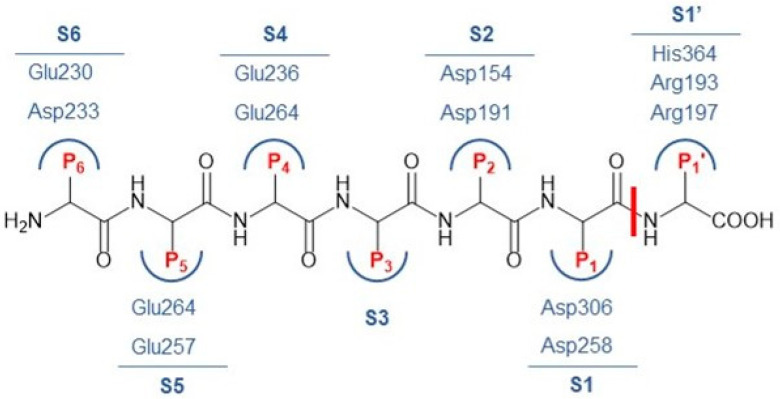
Representation of furin peptide substrates (P6-P1′, highlighted in red) with representative amino acids at each subsite (S6-S1′, highlighted in blue). The position where the peptide bond is cleaved by the catalytic triad is indicated by a red line.

The furin substrate binding site is extended and divided into several subsites, as represented in Figure 1. These subsites consist of amino acid residues that bind to individual residues of the peptide substrate. The C-terminal amino acid residue of the peptide substrate at the cleavage site is designated P1. The furin subsite of this residue is designated S1. The *N*-terminal amino acid of P1 is designated P2 and the subsite is designated S2. The amino acids that lie in the C-terminal of the cleaved bond are designated P1′, P2′, etc., and their subsites are named accordingly [1,3]. The residues in these subsites are mainly negatively charged, which explains why positively charged substrate ligands exhibit high inhibitory potency [3]. Residues surrounding the S1 subsite include Asp258 and Asp306, while the S2 subsite is delineated by residues Asp154 and Asp191. The S4 subsite is formed by Glu236 and Glu264, S5 by Glu257 and Glu264, and S6 by Glu230 and Asp233. There are no acidic residues in the vicinity of the S3 subsite and no basic residues in the region between the S1 and S6 subsites [10]. However, basic residues Arg193, Arg197, and His364 are found in the S1′ subsite [3].

**Figure 2:**
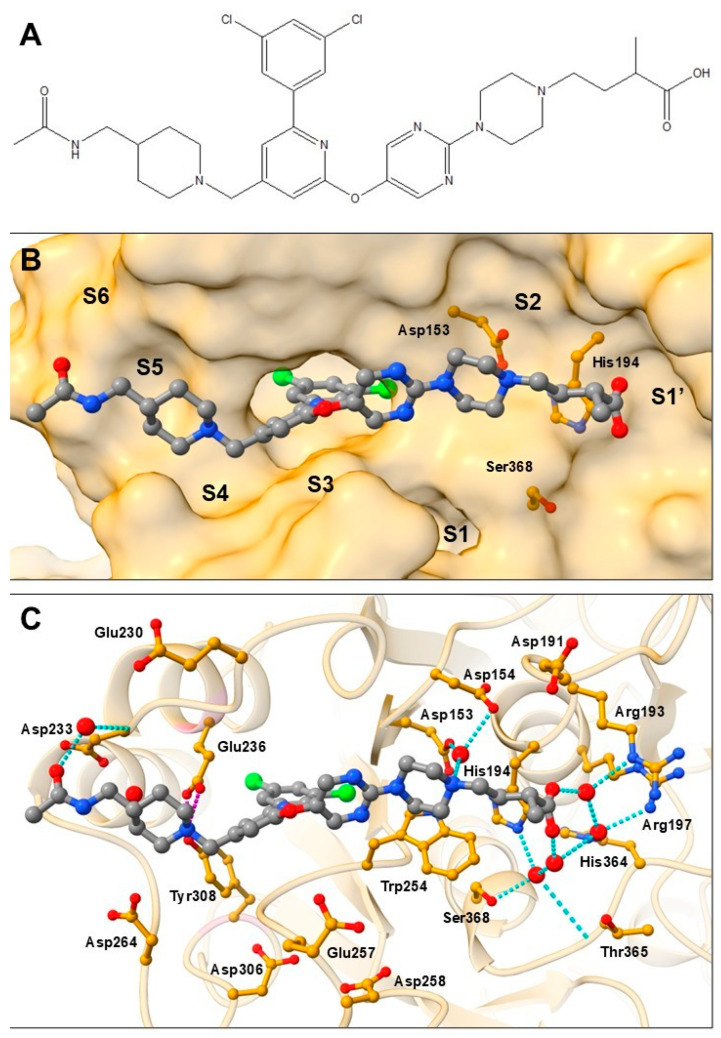
Binding mode of a BOS furin inhibitor representative I0Q (in gray ball-and-stick representation; PDB ID: 7QY2). (**A**) Chemical structure of BOS furin inhibitor representative I0Q (from PDB ID: 7QY2). (**B**) Binding of a BOS inhibitor to the relative position of furin subsites S1′–S6. The amino acid residues of the catalytic triad are shown in yellow ball-and-stick representation. (**C**) Interactions of a BOS inhibitor within the binding site of furin. Representative amino acid residues from each subsite and key binding residues are shown in a yellow ball-and-stick representation. The only direct interaction between inhibitor and furin, the salt bridge between the piperidine nitrogen and the Glu236 side chain, is depicted by magenta dashes. Water molecules involved in the inhibitor-protein-binding network are represented by red spheres and their interactions are denoted by cyan dashes.

The majority of furin inhibitors represent peptide-based or peptidomimetic inhibitors whose development has been halted due to their high molecular weight, low cell permeability, low cellular activity, lack of selectivity, metabolic or chemical instability, and cellular toxicity [3,11]. However, recent advances with furin involvement in SARS-CoV-2 infections have increased interest in this field and led to the development of non-peptide small-molecule furin inhibitors. The BOS series of compounds, chemically characterized as (3,5-dichlorophenyl)pyridine derivatives and developed by Boston Pharmaceuticals [12], has shown nanomolar inhibitory activity in cell-based assays and high in vitro efficacy. They were able to block SARS-CoV-2 S1/S2 cleavage by furin without imposing significant side effects [3,11,12,13] However, the clinical significance of furin as a validated target for the treatment of SARS-CoV-2 infection remains unclear and requires further biological studies both in vitro and in vivo. The BOS compound series contains several weakly basic moieties that can interact with negatively charged amino acid residues in the furin binding pocket, still providing bioavailability despite their weak basicity. Interestingly, the compounds also contain carboxylic acid fragments, indicating a different binding mode. The structure of representative BOS compound I0Q [14] is shown in Figure 2A.

Recently, the binding mode of these compounds was elucidated through the crystal structures of several BOS compounds in complex with furin, revealing a tight binding mechanism and a strong stabilization of the complex, along with several structural rearrangements of the furin substrate binding site [14,15]. The most significant rearrangement observed with these inhibitors is the flip of the Trp254 side chain of approximately 180°. This leads to the formation of an extended hydrophobic binding pocket into which the 3,5-dichlorophenyl moiety of the BOS compound I0Q can be inserted (Figure 2B). The conformational changes of Trp254 block access to the residues in the S1 and S2 pockets, preventing binding with these residues. This explains the observed slow tight binding kinetics and an induced-fit inhibition mechanism. Interestingly, the crystal structures of BOS compounds with furin showed that the ligand forms mainly water-mediated contacts with the furin binding site. The interaction network of the representative BOS compound I0Q [14] is shown in Figure 2C. The piperazine moiety has a protonated amine that forms an indirect interaction through water-mediated contact with the side chains of the catalytic Asp153 and Asp154. This interaction is important for efficacy but does not determine the overall binding. Compounds with negatively charged substituents on the piperazine are beneficial and exhibit a higher inhibitory potency, which is surprising due to the strong negative environment of the furin binding site. These residues form a water network between the negatively charged moiety of the inhibitor and the positively charged residues in the S1′ region of the furin binding site. The piperidine of the compound is also protonated and forms a salt bridge with the side chain of Glu236 in the S4 subsite. This is the only direct interaction of the compound not mediated by water. The piperidine substituents are mostly negatively charged residues that interact with amino acid residues in the S4–S6 subsite, such as Asp233 and Asp264, via a water-mediated network. However, it appears that strongly negatively charged moieties are less preferred due to electrostatic repulsion with the strongly negatively charged binding pocket [4].

**Figure 3:**
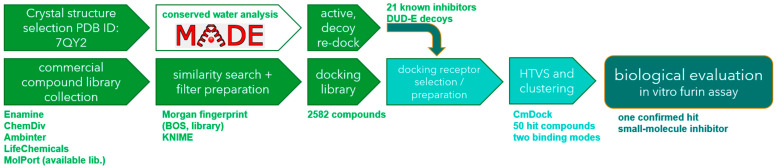
In silico protocol for identification of a new furin inhibitor.

With all the structural observations in mind, we report a water-aware screening study that takes advantage of the conserved water molecules and water-mediated bonds at the docking receptor preparation step to identify novel nonpeptidic small-molecule furin inhibitors. After an extensive virtual screening campaign, the top-scoring compounds forming key interactions were further biologically evaluated using an in vitro assay for their ability to inhibit furin cleavage of a protease substrate (Figure 3).

## 2. Results and Discussion

### 2.1. Compound Library Preparation

While the BOS compound series provides the most active inhibitors identified for furin with a new binding mode [3,11,12], the idea for the design of our library was to search for compounds that reside in a BOS-inhibitor-informed chemical space. Therefore, a virtual screening library was compiled from commercially available compounds from different vendors (Enamine, ChemDiv, Ambinter, LifeChemicals, MolPort). Two approaches were applied to assemble the library, both based on the BOS E1–E18 inhibitors (the same naming as in reference [3]; Table S1 in the Supporting Information), which exhibit furin inhibitory activity in vitro [3,12]. In the first approach, the Morgan fingerprint similarity search (0.3 Morgan fingerprint similarity cutoff) was used based on BOS E1-E18 inhibitors. In the second approach, the same BOS inhibitors were applied to design a physico–chemical similarity filter with the following parameters (Table S1 in Supporting Information): SMILES partition coefficient (SlogP) 4.4–6.2, average molecular weight (AMW) 557–678, topological polar surface area (TPSA) 94–134, hydrogen bond donors (HBD) 1–3, hydrogen bond acceptors (HBA) 8–10, and number of rotatable bonds 8–12. The compounds resulting from either approach were then combined in a joint library that displayed a wide selection of structures for further screening steps.

After library assembly, an in-house-developed Konstanz information miner (KNIME) protocol (Figure S1 in the Supporting Information) was employed to remove the known aggregators and rapid elimination of swill (REOS) structures to remove the reactive functional groups, followed by neutralization, the addition of hydrogen, and the optimization of geometry with the reaction database toolkit (RDKit; v2023.09.2) software nodes. This yielded 125 compounds for the first approach and 2457 for the second, resulting in a final library of 2582 compounds ready for subsequent virtual screening experiments [16].

### 2.2. Docking Receptor Preparation

BOS inhibitors have been found to interact with furin predominantly through a water-mediated network. Therefore, we performed an analysis of the available crystal structures of the furin–BOS inhibitor complexes to determine which water molecules are important for binding. To prepare the furin structure containing the new binding pocket for molecular docking, we used a structure with a small molecule bound in the new pocket with PDB ID: 7QY2. Moreover, we conducted a conserved water molecule analysis to determine which water molecules play a significant role in the structure. We used the ProBiS H2O and MADE methodologies [17,18,19]. Namely, we applied a set of five furin structures with similar small ligands bound in the newly discovered pocket with PDB IDs: 7LCU, 7QXY, 7QXZ, 7QY0, and 7QY1. These were superimposed onto the 7QY2 structure using TM-align [20]. The positions of water molecules in the superimposed structures were clustered by 3D-DBSCAN with the ε parameter set to 0.9 Å [21]. Within 5 Å of the ligand bound in 7QY2, we identified 21 clusters (see Supporting Information) where at least four out of the investigated six structures had a water molecule in a similar position. We applied a set of 21 known BOS active compounds (Table S2 in Supporting Information) to create a set of 1850 decoys using the Directory of Useful Decoys: Enhanced (DUD-E) [22]. We docked the actives and decoys to the receptor structures with different configurations of conserved water molecules present in an enrichment experiment.

We obtained the best enrichment (Table S3 in Supporting Information) when using a receptor structure with all 21 conserved water molecules (67% conservation level, MADE software, flexible water as per CmDock protocol) with the following parameters: a receiver-operating characteristic (ROC) area under the curve of 0.94, a Boltzmann-enhanced discrimination of receiver operating characteristic (BEDROC) (20) of 0.58, a BEDROC (80.5) of 0.38, an enrichment factor (EF) at 1% of 21.1 and an EF at 10% of 12.79. We also compared the results when the water molecules were included as part of the receptor structure and when treating them as stationary and rotatable solvents with CmDock software (v0.2.0). We found that treating the water as a rotatable solvent enhances enrichment at the cost of slowing down the calculations. Including the conserved water molecules results in a large increase in enrichment compared with the furin structure with no water included, which only gave a ROC area under curve (ROC AUC) of around 0.81. Therefore, in this manner, water information can be used effectively to enhance binding pose prediction and HTVS enrichments. It should be noted that upon hit identification in a HTVS campaign, individual binding poses can be further elaborated via molecular dynamics (MD) post-festum to aid in their mechanistic and structural modification studies.

### 2.3. Virtual Screening Experiment

After the virtual screening experiment using molecular docking as described in the Material and Methods section, the 50 highest-scoring compounds were selected according to the CmDock SCORE.INTER scoring function and their predicted binding modes were visually examined. We identified two general binding modes for the compounds, and the hits were classified into two clusters based on these binding modes. The first, larger cluster contained 40 hit compounds (Table S4 in Supporting Information, compounds **1**–**11** in Table 1), which were predicted to have a similar binding mode to the BOS inhibitors (Figure 2B,C). These compounds mainly occupied the S4, S5, and/or S6 subsites of the binding pocket, with their substituents also inserted into the newly formed hydrophobic pocket created by the rearrangement of Trp254. However, the binding of these compounds did not extend to the catalytic triad, leaving this side of the binding pocket unoccupied. The main interactions formed by the ligands in this cluster (Table S5 in Supporting Information) were hydrogen bonds with the carboxylate group of the Glu236 side chain, the only direct hydrogen bond identified for BOS inhibitors (Figure 2C), and the phenolic hydroxy group of the Tyr308 side chain from the S4 subdomain. Additionally, π–π interactions were observed with the aromatic side chains of Trp254 and Tyr308. Examining the water-mediated interactions of the ligands from cluster 1 with residues in the furin binding site, we found that the main water network involved a maximum of two water molecules interacting with the Asp233 main and side chains from subsite S6, the Gly255 main chain, the Glu257 main and side chains from subsite S5, the Asp264 side chain from subsites S4/S5, and the Ala267 main chain. As in our compounds, the water-mediated network with residues Asp233 and Asp264 was also identified for BOS furin inhibitors.

The second cluster of 10 hit compounds (Table S6 in Supporting Information, compounds **12**–**20** in Table 1) comprised the molecules whose predicted binding position resembles the binding of BOS inhibitors, with their substituents extending toward the catalytic triad in the S2 binding subsite (Figure 2B). Similar to the compounds in cluster 1, the interactions (Table S7 in Supporting Information) of this cluster involve hydrogen bonds formed mainly with the crucial carboxylate group of the Glu236 side chain of the S4 subdomain and π–π interactions with Tyr308. Water-mediated hydrogen bonds were again found with similar residues, namely the Gly255 main chain, the Glu257 main and side chains from subsite S5, the Asp264 side chain from subsite S4/S5, and the Ala267 main chain. Additionally, water-mediated networking was also found to a large extent in the S2 subsite, where most compounds form water-mediated hydrogen bonds with the side chain of the residue of the catalytic triad Asp153, the side chain of Asp154, and the Asp191 main chain. These interactions have been shown to be important for the biological activity of BOS inhibitors).

### 2.4. Biological Evaluation of Furin Protease Activity

Among the top 50 highest-scoring compounds (Tables S4 and S6 in Supporting Information) from molecular docking, the two compound clusters were subjected to contact analysis to select the compounds for biological evaluation on the furin protease assay. Our selection procedure focused on compounds that formed hydrogen bonds with the protein, particularly with the side chains of Glu236 and Tyr308, which were the most frequently observed hydrogen bond interactions (Tables S5 and S7 in Supporting Information). Therefore, we prioritized compounds from both clusters that formed hydrogen bonds with at least one of these residues. Additionally, we selected compounds from the second cluster that extended toward the catalytic triad and formed water-mediated interactions with residues Asp154 and/or Asp191 in the S2 subsite or the catalytic residue of Asp153. The selected compounds are presented in Table 1. To demonstrate their novel structures, their similarity to the known BOS inhibitors E1–E18 was calculated (Table S8 in the Supporting Information). Our final selection criteria thus included eleven commercially available compounds from cluster 1 (compounds **1**–**11**) that formed hydrogen bonds with Glu236 and/or Tyr308 and nine commercially available compounds from cluster 2 (compounds **12**–**20**) that formed either direct hydrogen bonds with Glu236 and Tyr308 or water-mediated interactions with key residues in the S2 subsite (Asp154 and/or Asp191) or the catalytic key residue of Asp153. The ability of the 20 selected compounds to inhibit furin activity was then assessed in an in vitro furin protease assay, measuring their ability to prevent cleavage of the furin protease substrate. The results are summarized in Table 1.

After the preliminary screening, compound **4** (MolPort-000-500-670, ZINC000097968827): *N*-[4-(1,3-thiazol-2-ylaminosulfonyl)phenyl]-3-{(E)-5-[(2-methoxyphenyl)methylene]-4-oxo-2-thioxo-1,3-thiazolidin-3-yl}propionamide was found to inhibit furin protease by 86% at a concentration of 100 μM. Subsequent IC_50_ measurements supported this observation with an IC_50_ value of 17.6 ± 2.3 μM (Table 1). The 2-sulfanylidene-1,3-thiazolidin-4-one (rhodanine) scaffold of the hit compound was flagged as a potential frequent hitter or a pan assay interference compound (PAINS), and this should be noted in further research [23]. As the chemical space of furin inhibitors is small and only a few compounds are reported, we opted towards reporting this compound and we believe it can serve as an optimization starting point or probe compound. Namely, similar scaffold compounds have recently shown high selectivity and potent activity toward their targets [24,25]. Moreover, as a proof of concept, derivatives with antitumor, anti-inflammatory, antidiabetic, and other therapeutic properties have been described in the literature as starting points for further optimization [25]. Therefore, we believe that our identified hit, compound **4**, is of interest as no similar scaffold is currently described as a furin inhibitor or reported in ChEMBL database.

In a further examination of close structural analogs to the hit **4**, a related compound, *N*-[4-(1,3-thiazol-2-ylaminosulfonyl)phenyl]-3-[(Z)-5-phenylmethylene-4-oxo-2-thioxo-1,3-thiazolidin-3-yl]propionamide (CCT021812), was reported as an inhibitor of the protein methyltransferase SET7/9 [26]. Protein methyltransferases are enzymes responsible for the methylation of histones that regulate transcription. The compound CCT021812 proved to be a promising hit for the inhibition of SET7/9 and showed confirmed activity in various in vitro assays. Regarding concerns about its potential as a frequent hitter due to the 2-sulfanylidene-1,3-thiazolidin-4-one scaffold, CCT021812 showed consistent activity in all assays. Considering the similarity to our hit compound, it can be speculated that our compound could also be useful for the development of protein methyltransferase inhibitors. Moreover, the moderate activity of CCT021812 leaves space for further structural optimization where our hit compound **4** could be of use [26].

**Figure 4:**
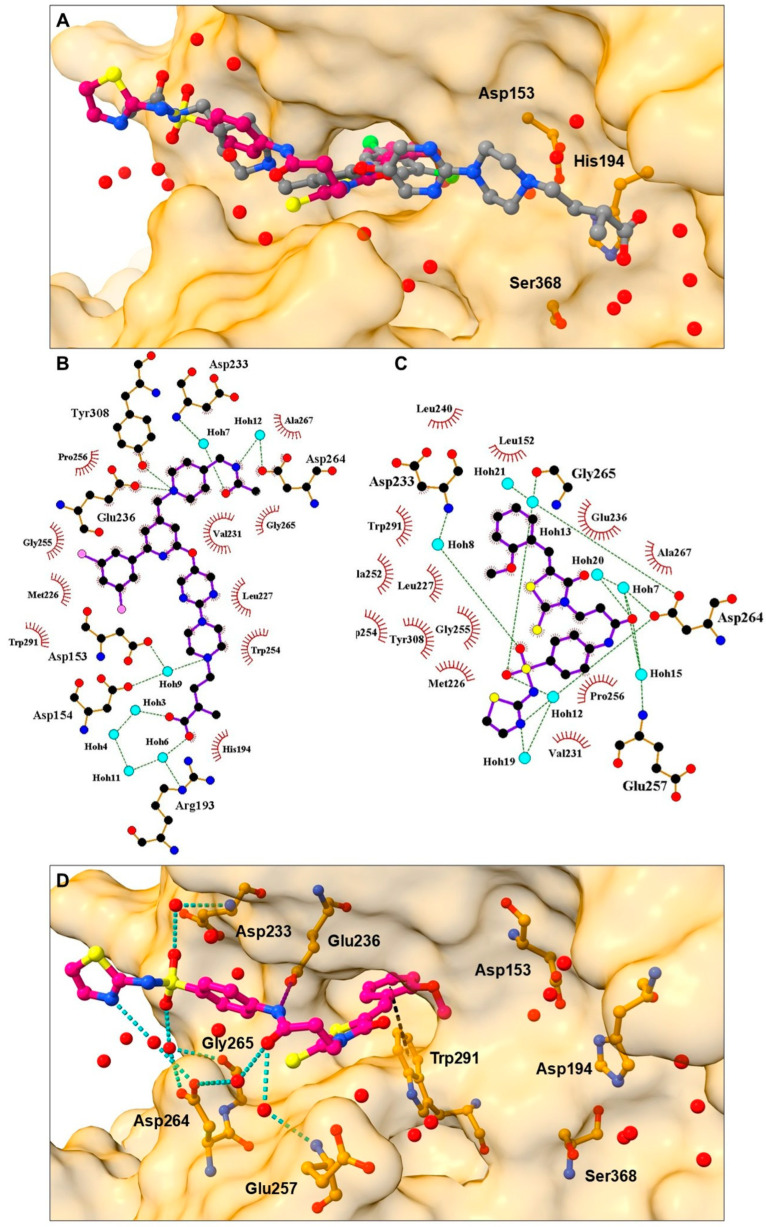
(**A**) Superposition of hit compound **4** (purple color with emphasized hetero-elements in stick model) and crystal ligand I0Q from PDB ID: 7QY2 in gray-colored stick model. (**B**) Crystal ligand I0Q 2D interaction diagram with the protein. (**C**) Hit compound **4** 2D interaction diagram with the protein. (**D**) The binding mode of the most active hit compound **4** from cluster 1 (purple color with emphasized hetero-elements in stick model) in the active site. Amino acid residues in the active site are labeled with emphasized active site surface in a transparent orange color.

Comparing the binding mode of the most active compound **4** scaffold and the binding mode of BOS inhibitor I0Q (Figure 4A) [14], we can observe that while exhibiting a similar binding mode, compound **4** displays an alternative interacting pattern (Figure 4B–D). While retaining key hydrogen bond interaction with Glu236 and hydrophobic contacts with Val231 and Ala267 through its 1,3-thiazol-2-ylaminosulfonyl moiety, the compound protrudes into the pocket bordered by Leu152, 240, Met226, and Tyr308 with its terminal o-Methoxyphenyl terminal moiety without stretching towards a hydrophilic catalytic site. The ligand effectively utilizes a conserved water-laden receptor and engages in two conserved water-bridges to Asp233 and Glu236. Compound **4** thus displays hydrogen bonds towards Asp233 and Glu236; hydrophobic interactions with Leu152, Leu240, Ala252, and Tyr308; along with π-stacking with Trp291 with the aforementioned water-mediated H-bonds (Figure 4D).

## 3. Material and Methods

### 3.1. Molecular Docking Calculations

We performed molecular docking using in-house-developed CmDock software (v. 0.2.0; https://gitlab.com/Jukic/cmdock, accessed on 1 December 2024), which represents a fork of RxDock/rDock with modern tool additions, optimizations, and adaptions for modern hardware and software [27]. The receptor structure was prepared by removing all non-protein atoms and alternative state residues from the 7QY2 structure and then adding the conserved water molecules from our MADE study. The Prepare Protein Wizard in Maestro v. 12.6 (Release 2020-4, Schrödinger, LLC, New York, NY, USA) was applied to prepare the protein structure for subsequent docking experiments. Due to demanding (cpu/hour) calculations via the inclusion of fully rotatable water molecules in the receptor definition, the experiments were performed on the VEGA HPC system. We applied the experimental co-crystallized ligand to define the search grid of 10 Å around the reference ligand heavy atoms (ligand I0Q; 601; A; from PDB ID: 7QY2). The crystal structure with PDB ID: 7QY2 was downloaded from the RCSB Protein Data Bank (PDB). The docking site was defined as having a total volume 7223.25 Å^3^ with Cavity #1 (Size = 57,786 points; Vol = 7223.25 A^3^; Min = (38, −49, −26.5); Max = (65, −18.5, 15); Center = (52.3768, −34.5672, −4.78932); Extent = (27, 30.5, 41.5)).

Twenty-one explicit water molecules were considered during molecular docking via the SECTION SOLVENT parameter in the CmDock docking protocol. Solvent was always considered (OCCUPANCY parameter 1.0) and sampled as free (TRANS MODE FREE parameter). With 100 runs utilizing DOCK.prm settings, we applied a sample technique that included three stages of genetic algorithm search, low-temperature Monte Carlo, and simplex minimization phases, as well as scoring using the rDOCK (SF3) scoring function [27]. The sampling and scoring methodologies of CmDock were previously validated against protein [27,28] and RNA [27,29] targets, and their performance was superior to similar open-source software [30]. Moreover, we also performed a validation re-docking study on our prepared system (PDB ID: 7QY2) before commencing with HTVS. We successfully re-docked the 7QY2 I0Q small-molecule ligand with an RMSD of 0.93 Å (further re-docking info in Supporting Information, Table S9, Figure S2).

The calculated docking poses were sorted according to the CmDock SCORE.INTER scoring function and their predicted binding modes were examined using Maestro 12.6 (release 2020-4, Schrödinger, LLC, New York, NY, USA).

### 3.2. Furin Protease Assay

The compounds selected as potential inhibitors for subsequent biological evaluation were purchased from MolPort Inc. (Riga, Latvia). The Furin Protease Assay Kit was obtained from BPS Bioscience Inc. (San Diego, CA, USA) to measure furin protease activity in the presence of the investigated compounds. The assay was performed on 96-well plates according to the manufacturer’s protocol. Furin was initially diluted to 0.5 ng/μL in an assay buffer and 50 μL of it was added to the wells. Potential furin inhibitors dissolved in DMSO (Sigma-Aldrich, St. Louis, MO, USA) were then diluted with assay buffer to a concentration 10-fold higher than the final concentration and 10 μL was added to the furin-containing wells. Fluorogenic furin protease substrate was diluted in assay buffer to a concentration of 5 μM, and 40 μL of it was added to the wells to start the reaction. The reaction took place for 30 min at room temperature. Then, the fluorescence intensity of the protease substrate was measured using microplate reader (excitation, 380 nm; emission, 460 nm). The assay buffer was applied as a blank and the protease substrate as a positive control. Moreover, the known furin inhibitor chloromethylketone, supplied with the assay kit, was used as an inhibitor control at a final concentration of 0.05 μM. Preliminary screening of the compounds was performed at two final inhibitor concentrations of 100 and 10 μM, and IC_50_ values were determined at different inhibitor concentrations. The inhibition percentage was calculated using the following formula:

$$Furin\;activity\;inhibition=100\%-\left(\frac{\left(I-B\right)}{\left(P-B\right)}*100\%\right)$$

where I is the fluorescence intensity measured in the wells containing the compounds, B is the fluorescence intensity measured in the blank wells, and P is the fluorescence intensity in the positive control wells. The value of the half-maximal inhibitory concentration (IC_50_) was calculated using a nonlinear regression function with GraphPad Prism software (v6.0, GraphPad Software, La Jolla, CA, USA). The experiments to determine the percentage of inhibition were performed in duplicate, while the dose-dependent experiments to determine the IC_50_ values were performed in triplicate.

## 4. Conclusions

In this present work, we identified a non-peptidic small-molecule inhibitor of furin protease substrate cleavage with a 1,3-thiazol-2-ylaminosulfonyl scaffold that was previously unknown in the chemical space of furin inhibitors. As small molecules, these compounds may have typical advantages over peptide inhibitors, e.g., metabolic and structural stability, lower immunogenicity, cell permeability, bioavailability, and, most importantly, synthetic availability and modification potential. Compound **4**, *N*-[4-(1,3-thiazol-2-ylaminosulfonyl)phenyl]-3-{(E)-5-[(2-methoxyphenyl)methylene]-4-oxo-2-thioxo-1,3-thiazolidin-3-yl}propionamide, exhibited an IC_50_ of 17.58 ± 2.29 μM in the furin protease in vitro inhibition assay. Docking studies show that although the compound has a similar binding mode to BOS inhibitors, it does not bind to the binding pocket where the catalytic triad is located. This provides an opportunity for structural optimization, allowing us to target unoccupied binding pockets to increase binding affinity and potentially improve the activity of the compound, as well as exchange the central heterocyclic core with bioisosteres like imidazolidine-2,4-dione. Therefore, compound **4** may be of interest to the medicinal chemistry community for further structural evolution, biological selectivity studies, and in vitro and cell-based assays with the aim of discovering and optimizing novel small-molecule furin inhibitors. The remainder of the reported set can be effectively utilized as a source of experimental decoys for furin protease inhibitor studies.

## Figures and Tables

**Table 1:** Twenty docking hit compounds subjected to biological evaluation.

Cmpd	Structure	SCORE.INTER Score	Biological Activity ^2^ [IC_50_; μM] ^3^ or % Inhibition ^4^
**CLUSTER 1** ^1^			
**1** MolPort-003-017-240	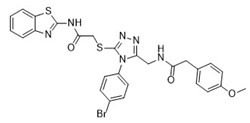	−48.1028	NA
**2** Amb3424462	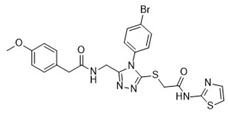	−43.9082	NA
**3** Amb8607920	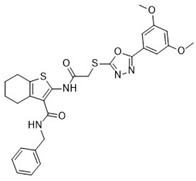	−41.3367	NA
**4** MolPort-000-500-670	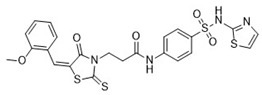	−41.191	17.58 ± 2.29 85.52% ± 0.47
**5** Amb3498025	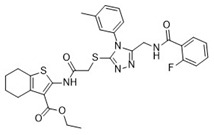	−40.6428	NA
**6** MolPort-000-805-473	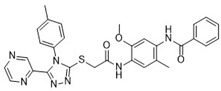	−40.4531	NA
**7** Molport-007-869-358	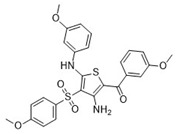	−40.3534	28.06 ± 6.17%
**8** MolPort-000-757-950	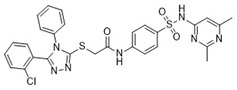	−39.6437	NA
**9** Molport-028-876-901	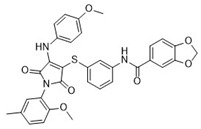	−38.9761	35.86 ± 2.26%
**10** MolPort-003-018-758	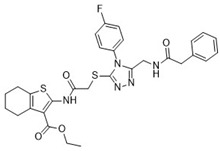	−38.8141	NA
**11** Amb3422793	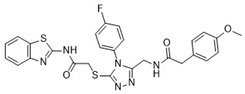	−38.8028	NA
**CLUSTER 2** ^1^			
**12** MolPort-003-054-063	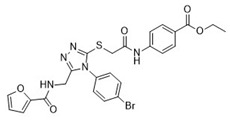	−46.3925	NA
**13** MolPort-007-908-874	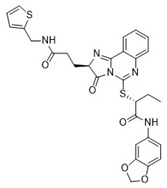	−42.0558	NA
**14** Amb3476897	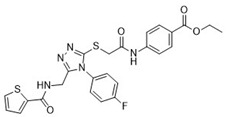	−41.4865	NA
**15** K250-0182	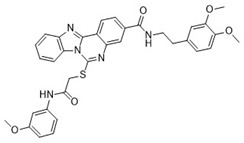	−41.1365	NA
**16** MolPort-000-802-159	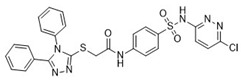	−40.079	NA
**17** MolPort-028-887-472	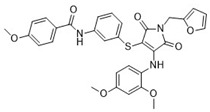	−39.7724	NA
**18** MolPort-001-969-537	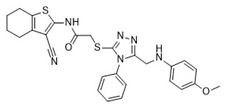	−39.4484	NA
**19** MolPort-003-052-514	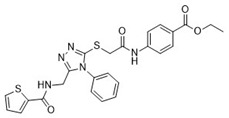	−39.4001	NA
**20** MolPort-003-071-523	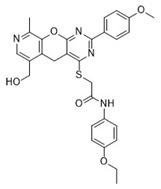	−38.6347	NA

^1^ Cluster 1: compounds **1**–**11**; cluster 2: compounds **12**–**20**. ^2^ NA means that the compound showed no activity at the concentrations assayed. ^3^ Experiments to determine the IC_50_ values were performed in triplicate. ^4^ % of inhibition of the furin activity obtained at the compound concentration of 100 μM and presented as mean ± SD percentage of two measurements.

## Data Availability

Data is contained within the article or Supplementary Material.

## Supplementary Materials

The following supporting information can be downloaded at: https://www.mdpi.com/article/10.3390/ph18020273/s1, Table S1: Calculated RDKit molecular descriptors for known BOS furin inhibitors, Table S2: Compounds in cluster 1, Table S3: Compounds in cluster 2, Table S4: Cluster 1 analysis, Table S5: Cluster 2 analysis; Table S6: Top scoring compounds in cluster 2; Table S7: The table shows the number and percentage of ligands out of a total of 10 from cluster 2 that exhibit a specific type of interaction with amino acid residues in the furin binding site; Table S8: Percentage similarity of the top-scored compounds in clusters 1 and 2 with the known BOS inhibitors from Table S1; Table S9: Re-docking validation experiment (3 top poses examined); Figure S1: KNIME workflow for agregator and REOS fitering; Figure S2: Re-docking of the 7QY2 I0Q small-molecule ligand with an RMSD of 0.93 Å. Reference [31] is cited in the supplementary materials.
