# Supplementary material for: Identification of Furin Protease Small-Molecule Inhibitor with a 1,3-Thiazol-2-ylaminosulfonyl Scaffold
Source: Pharmaceuticals (Basel). 2025 Feb 19;18(2):273. doi: 10.3390/ph18020273 (PMC11859198; doi:10.3390/ph18020273)
Supplement: Supplementary file 1 [file pharmaceuticals-18-00273-s001.zip › pharmaceuticals-3463649-supplementary.pdf]

Supporting information

For

Identification of Furin Protease Small-Molecule Inhibitor with a 1,3-thiazol-2-ylaminosulfonyl Scaffold

Anja Kolarič [1], Vid Ravnik [1], Sara Štumpf Horvat [1], Marko Jukič [1,3 *], Urban Bren [1,2,3 *]

[1] Laboratory of Physical Chemistry and Chemical Thermodynamics, Faculty of Chemistry and Chemical Engineering, University of Maribor, Smetanova 17, SI-2000 Maribor, Slovenia

[2] IOS, Institute of Environmental Protection and Sensors, Beloruska ulica 7, SI-2000 Maribor, Slovenia

[3] Faculty of Mathematics, Natural Sciences and Information Technologies, University of Primorska, Glagoljaška 8, SI-6000 Koper, Slovenia

Table S1. Calculated RDKit molecular descriptors for 18 known BOS furin inhibitors [1,2,3] using KNIME and corresponding descriptor thresholds established for filtering commercial libraries to create a virtual screening library.

		SlogP	AMW	TPSA	NumHBD	NumHBA	NumRotatableBonds
BOS inhibitor[a]	E1	4.797	627.573	111.13	2	8	10
	E2	5.188	641.6	111.13	2	8	11
	E3	4.757	675.683	107.97	1	9	11
	E4	4.705	613.59	94.06	2	8	10
	E5	4.980	656.615	123.16	3	8	11
	E6	5.076	571.509	94.92	1	8	8
	E7	5.466	585.536	94.92	1	8	8
	E8	4.734	557.482	103.71	2	8	8
	E9	5.076	571.509	94.92	1	8	8
	E10	5.466	585.536	94.92	1	8	8
	E11	5.219	583.52	94.92	1	8	8
	E12	6.102	613.59	94.92	1	8	10
	E13	4.881	677.655	129.06	1	10	12
	E14	4.827	615.562	115.15	2	9	10
	E15	4.439	601.535	115.15	2	9	10
	E16	4.583	642.588	124.02	2	9	11
	E17 (I0Q)[b]	5.219	670.642	124.02	2	9	12
	E18	4.802	658.587	133.25	2	10	11
**Descriptor limits**		4,4-6,2	557-678	94-134	1-3	8-10	8-12

[a] The designation is the same as in reference [1]. [b] Compound from reference [2].

Conserved water definition in CmDock .prm file:

SECTION SOLVENT

FILE important_waters.pdb

END_SECTION

important_waters.pdb:

HETATM 1 OW HOH W 1 40.385 -41.648 -17.330 1.00 2.00 O

HETATM 2 H1 HOH W 1 40.163 -42.096 -18.149 1.00 2.00 H

HETATM 3 H2 HOH W 1 40.846 -42.255 -16.747 1.00 2.00 H

HETATM 4 OW HOH W 2 39.692 -32.244 -8.966 1.00 2.00 O

HETATM 5 H1 HOH W 2 39.067 -31.684 -9.431 1.00 2.00 H

HETATM 6 H2 HOH W 2 39.943 -32.980 -9.528 1.00 2.00 H

HETATM 7 OW HOH W 3 42.229 -40.131 -14.894 1.00 2.00 O

HETATM 8 H1 HOH W 3 42.756 -39.520 -15.415 1.00 2.00 H

HETATM 9 H2 HOH W 3 42.104 -39.774 -14.011 1.00 2.00 H

HETATM 10 OW HOH W 4 40.001 -39.690 -13.300 1.00 2.00 O

HETATM	11	H1	HOH W	4	39.811	-39.042	-12.617	1.00	2.00	H
HETATM	12	H2	HOH W	4	39.907	-39.282	-14.164	1.00	2.00	H
HETATM	13	OW	HOH W	5	49.376	-28.606	2.640	1.00	2.00	O
HETATM	14	H1	HOH W	5	49.571	-29.349	2.065	1.00	2.00	H
HETATM	15	H2	HOH W	5	49.451	-27.788	2.143	1.00	2.00	H
HETATM	16	OW	HOH W	6	42.014	-37.499	-17.816	1.00	2.00	O
HETATM	17	H1	HOH W	6	42.809	-37.795	-18.264	1.00	2.00	H
HETATM	18	H2	HOH W	6	41.796	-36.610	-18.104	1.00	2.00	H
HETATM	19	OW	HOH W	7	52.348	-35.667	-0.149	1.00	2.00	O
HETATM	20	H1	HOH W	7	52.450	-36.072	0.716	1.00	2.00	H
HETATM	21	H2	HOH W	7	51.954	-36.299	-0.754	1.00	2.00	H
HETATM	22	OW	HOH W	8	54.818	-26.826	1.054	1.00	2.00	O
HETATM	23	H1	HOH W	8	54.727	-27.587	1.633	1.00	2.00	H
HETATM	24	H2	HOH W	8	54.563	-26.031	1.528	1.00	2.00	H
HETATM	25	OW	HOH W	9	49.848	-37.327	-5.671	1.00	2.00	O
HETATM	26	H1	HOH W	9	49.092	-37.445	-6.251	1.00	2.00	H
HETATM	27	H2	HOH W	9	50.157	-38.184	-5.369	1.00	2.00	H
HETATM	28	OW	HOH W	10	44.871	-33.578	-13.282	1.00	2.00	O
HETATM	29	H1	HOH W	10	44.432	-33.806	-12.459	1.00	2.00	H
HETATM	30	H2	HOH W	10	44.921	-32.623	-13.364	1.00	2.00	H
HETATM	31	OW	HOH W	11	46.842	-33.201	-15.407	1.00	2.00	O
HETATM	32	H1	HOH W	11	46.442	-33.739	-16.094	1.00	2.00	H
HETATM	33	H2	HOH W	11	47.064	-33.754	-14.655	1.00	2.00	H
HETATM	34	OW	HOH W	12	54.279	-33.081	3.582	1.00	2.00	O
HETATM	35	H1	HOH W	12	53.761	-33.540	2.917	1.00	2.00	H
HETATM	36	H2	HOH W	12	54.523	-33.695	4.279	1.00	2.00	H
HETATM	37	OW	HOH W	13	51.628	-32.320	5.598	1.00	2.00	O
HETATM	38	H1	HOH W	13	51.281	-32.893	4.911	1.00	2.00	H
HETATM	39	H2	HOH W	13	51.843	-32.843	6.374	1.00	2.00	H
HETATM	40	OW	HOH W	14	39.769	-39.050	-17.160	1.00	2.00	O
HETATM	41	H1	HOH W	14	39.342	-39.043	-16.300	1.00	2.00	H
HETATM	42	H2	HOH W	14	39.811	-38.155	-17.504	1.00	2.00	H

HETATM	43	OW	HOH W	15	52.359 -37.483 -1.861 1.00 2.00	O
HETATM	44	H1	HOH W	15	51.881 -38.046 -1.248 1.00 2.00	H
HETATM	45	H2	HOH W	15	52.449 -36.604 -1.485 1.00 2.00	H
HETATM	46	OW	HOH W	16	46.038 -38.640 -5.513 1.00 2.00	O
HETATM	47	H1	HOH W	16	45.589 -37.885 -5.901 1.00 2.00	H
HETATM	48	H2	HOH W	16	46.230 -39.285 -6.197 1.00 2.00	H
HETATM	49	OW	HOH W	17	55.626 -29.834 -2.297 1.00 2.00	O
HETATM	50	H1	HOH W	17	56.304 -30.336 -1.839 1.00 2.00	H
HETATM	51	H2	HOH W	17	55.354 -30.308 -3.086 1.00 2.00	H
HETATM	52	OW	HOH W	18	47.712 -38.790 -7.885 1.00 2.00	O
HETATM	53	H1	HOH W	18	48.021 -38.305 -7.116 1.00 2.00	H
HETATM	54	H2	HOH W	18	47.494 -39.690 -7.630 1.00 2.00	H
HETATM	55	OW	HOH W	19	55.629 -33.697 5.771 1.00 2.00	O
HETATM	56	H1	HOH W	19	55.255 -32.921 5.347 1.00 2.00	H
HETATM	57	H2	HOH W	19	55.807 -34.370 5.110 1.00 2.00	H
HETATM	58	OW	HOH W	20	54.565 -33.910 -1.385 1.00 2.00	O
HETATM	59	H1	HOH W	20	54.545 -34.780 -0.979 1.00 2.00	H
HETATM	60	H2	HOH W	20	53.908 -33.347 -0.969 1.00 2.00	H
HETATM	61	OW	HOH W	21	52.639 -31.386 7.827 1.00 2.00	O
HETATM	62	H1	HOH W	21	52.540 -31.857 8.658 1.00 2.00	H
HETATM	63	H2	HOH W	21	52.313 -30.488 7.927 1.00 2.00	H

Table S2. A set of known BOS compounds, used to generate decoys.

Cmpd	Molecule (Canonical)
Cmpd 1 [a]	ClC1=CC(C2=CC(CN3CCC(CNC(C)=O)CC3)=CC(OC4=CN=C(N5CCN(CCOC)CC5)C=C4)=N2)=C C(
Cmpd 4 [a]	ClC1=CC(C2=CC(CN3CCC(CNC(C)=O)CC3)=CC(OC4=CN=C(N5CCN(CCC(O)=O)CC5)C=C4)=N 2)=
Cmpd 5 [a]	ClC1=CC(C2=CC(CN3CCC(OCC(O)=O)C3)=CC(OC4=CN=C(N5CCN(C)CC5)N=C4)=N2)=CC(Cl) =C1
E1	CC(=O)NCC1CCN(Cc2cc(Oc3ccc(N4CCN(CC(=O)O)CC4)nc3)nc(-c3cc(Cl)cc(Cl)c3)c2)CC1
E2	CC(=O)NCC1CCN(Cc2cc(Oc3ccc(N4CCN(CCC(=O)O)CC4)nc3)nc(-c3cc(Cl)cc(Cl)c3)c2)CC1
E3	CC(=O)NCC1CCN(Cc2cc(Oc3ccc(N4CCN(CCS(C)(=O)=O)CC4)nc3)nc(- c3cc(Cl)cc(Cl)c3)c2)CC1
E4	CC(=O)NCC1CCN(Cc2cc(Oc3ccc(N4CCN(CCO)CC4)nc3)nc(-c3cc(Cl)cc(Cl)c3)c2)CC1
E5	CNC(=O)NCC1CCN(Cc2cc(Oc3ccc(N4CCN(CCC(=O)O)CC4)nc3)nc(-c3cc(Cl)cc(Cl)c3)c2)CC1
E6	CN1CCN(c2cnc(Oc3cc(CN4CCC(CC(=O)O)CC4)cc(-c4cc(Cl)cc(Cl)c4)n3)cn2)CC1

E7	CN1CCCN(c2cnc(Oc3cc(CN4CCC(CC(=O)O)CC4)cc(-c4cc(Cl)cc(Cl)c4)n3)cn2)CC1
E8	O=C(O)CC1CCN(Cc2cc(Oc3cnc(N4CCNCC4)nc3)nc(-c3cc(Cl)cc(Cl)c3)c2)CC1
E9	CN1CCN(c2ncc(Oc3cc(CN4CCC(CC(=O)O)CC4)cc(-c4cc(Cl)cc(Cl)c4)n3)cn2)CC1
E10	CN1CCCN(c2ncc(Oc3cc(CN4CCC(CC(=O)O)CC4)cc(-c4cc(Cl)cc(Cl)c4)n3)cn2)CC1
E11	O=C(O)CC1CCN(Cc2cc(Oc3cnc(N4CCN5CCC4C5)nc3)nc(-c3cc(Cl)cc(Cl)c3)c2)CC1
E12	CC(CC(=O)O)CC1CCN(Cc2cc(Oc3cnc(N4CCN(C)CC4)nc3)nc(-c3cc(Cl)cc(Cl)c3)c2)CC1
E13	CS(=O)(=O)CCCN1CCN(c2ncc(Oc3cc(CN4CCC(CC(=O)O)CC4)cc(-c4cc(Cl)cc(Cl)c4)n3)cn2)CC1
E14	C[C@@H](O)CN1CCN(c2ncc(Oc3cc(CN4CCC(CC(=O)O)CC4)cc(-c4cc(Cl)cc(Cl)c4)n3)cn2)CC1
E15	O=C(O)CC1CCN(Cc2cc(Oc3cnc(N4CCN(CCO)CC4)nc3)nc(-c3cc(Cl)cc(Cl)c3)c2)CC1
E16	CC(=O)NCC1CCN(Cc2cc(Oc3cnc(N4CCN(CCC(=O)O)CC4)nc3)nc(-c3cc(Cl)cc(Cl)c3)c2)CC1
E17	CC(=O)NCC1CCN(Cc2cc(Oc3cnc(N4CCN(CCC(C)C(=O)O)CC4)nc3)nc(-c3cc(Cl)cc(Cl)c3)c2)CC1
E18	COC(=O)NCC1CCN(Cc2cc(Oc3cnc(N4CCN(CCC(=O)O)CC4)nc3)nc(-c3cc(Cl)cc(Cl)c3)c2)CC1

[a] BOS compounds from reference [2].

Table S3. Receptor water identification enrichment experiment.

Receptor	ROC_AUC	BEDROC (20)	BEDROC (80.5)	EF1%	EF5%	EF10%	Dock_Time [s]
Water_consv_67	0,943	0,577	0,377	21,090	12,789	8,095	3478,7
Water_consv_80	0,936	0,463	0,200	11,717	10,894	7,857	1726,0
Water_rec_consv_80	0,931	0,493	0,215	11,717	11,841	7,619	107,6
Water_consv_100	0,923	0,391	0,147	9,373	8,999	7,143	1060,6
Waters_1234_rec_consv_67	0,922	0,465	0,278	18,747	9,473	7,143	774,1
Water_rec_consv_100	0,907	0,395	0,186	14,060	9,473	6,667	110,0
Water_rec_consv_67	0,889	0,371	0,192	9,373	8,052	5,476	115,0
Waters_12	0,881	0,269	0,077	4,687	4,263	5,714	522,3
Waters_13	0,879	0,217	0,043	2,343	4,263	4,524	509,4
Waters_123	0,876	0,212	0,046	0,000	3,789	4,286	622,9
Waters_rec_13	0,875	0,218	0,047	2,343	3,789	4,286	105,0
Waters_14	0,872	0,197	0,025	0,000	2,842	5,000	495,3
Waters_124	0,871	0,155	0,007	0,000	1,895	3,810	613,6
Waters_134	0,869	0,228	0,047	2,343	3,789	5,476	603,3
Waters_1	0,868	0,219	0,065	4,687	3,316	4,286	433,0
Waters_1234	0,859	0,214	0,065	2,343	3,789	4,286	717,9
Waters_rec_1	0,852	0,181	0,032	0,000	3,789	3,810	108,2
Waters_rec_14	0,848	0,181	0,031	2,343	3,316	3,810	106,4
Waters_rec_12	0,845	0,161	0,033	2,343	1,421	3,571	104,1
Waters_rec_134	0,836	0,179	0,022	0,000	3,789	3,571	104,3
Waters_rec_3	0,825	0,149	0,022	0,000	2,368	2,619	103,7
Waters_4	0,822	0,183	0,067	2,343	3,316	2,857	138,4
Waters_3	0,813	0,141	0,012	0,000	1,895	3,333	142,7
No_Water	0,813	0,142	0,016	0,000	2,368	3,571	107,0
Waters_rec_124	0,810	0,159	0,038	2,343	2,368	3,571	104,5
Waters_2	0,801	0,146	0,035	2,343	2,368	2,619	146,3
Waters_23	0,797	0,135	0,010	0,000	1,895	3,095	185,4
Waters_rec_34	0,797	0,162	0,051	2,343	2,842	3,095	105,1
Waters_rec_4	0,796	0,105	0,004	0,000	0,947	2,857	103,6
Waters_rec_23	0,795	0,108	0,006	0,000	1,895	2,619	102,9
Waters_rec_2	0,794	0,071	0,003	0,000	0,474	1,429	104,8
Waters_34	0,793	0,142	0,040	2,343	3,316	2,619	181,1

Waters_rec_123	0,793	0,084	0,003	0,000	0,474	1,667	106,2
Waters_rec_1234	0,791	0,071	0,004	0,000	0,474	1,429	107,8
Waters_234	0,776	0,149	0,054	2,343	1,895	3,095	235,6
Waters_rec_234	0,771	0,107	0,020	2,343	0,947	2,381	104,3

Table S4. Top scoring compounds in cluster 1.

Cmpd	Structure	SCORE.INTER Score
MolPort-004-932-778	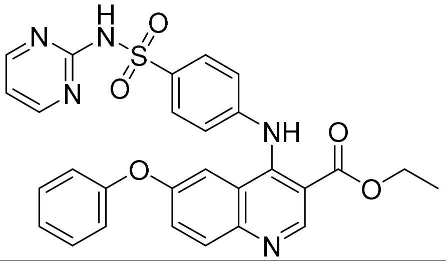	-50.6371
**1** MolPort-003-017-240	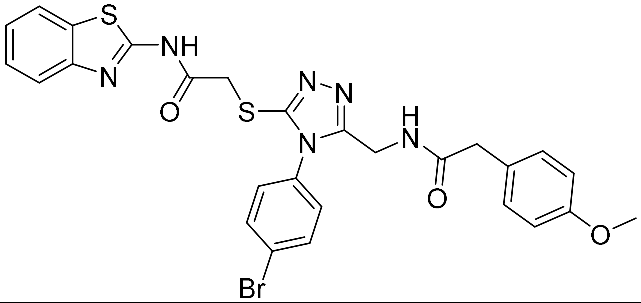	-48.1028
**2** Amb3424462	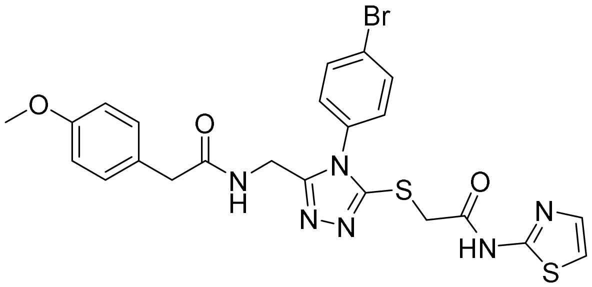	-43.9082
Amb395359	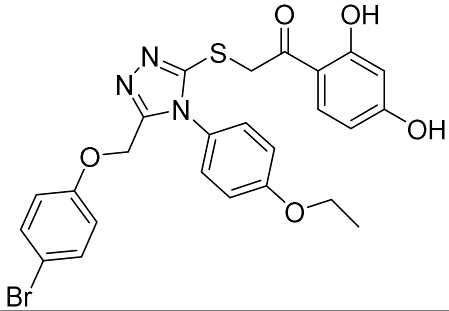	-43.1107
MolPort-002-946-465	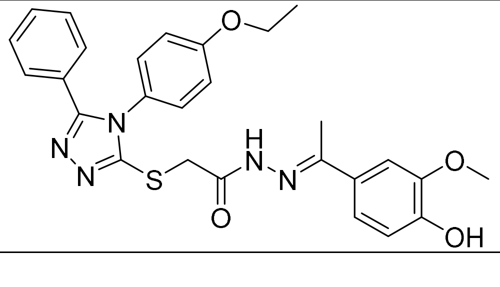	-42.7188

Amb3424381	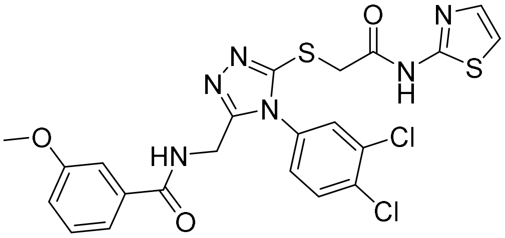	-42.6481
MolPort-028-893-466	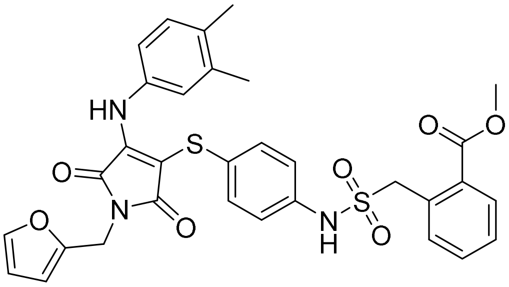	-42.1846
MolPort-000-440-835	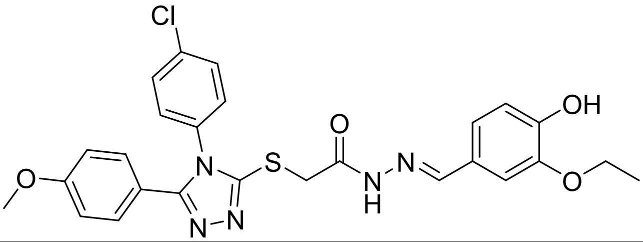	-41.9561
MolPort-000-445-638	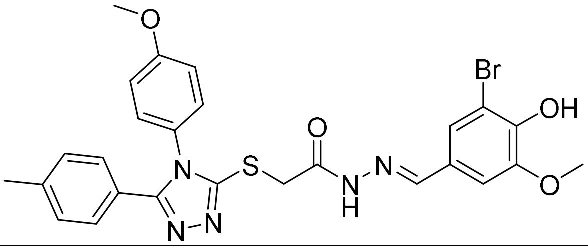	-41.4369
**3** Amb8607920	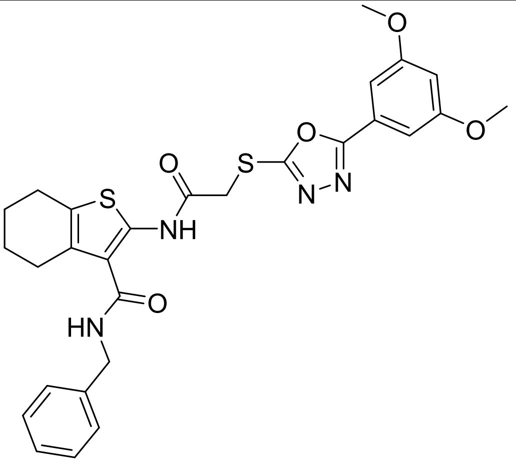	-41.3367
**4** MolPort-000-500-670	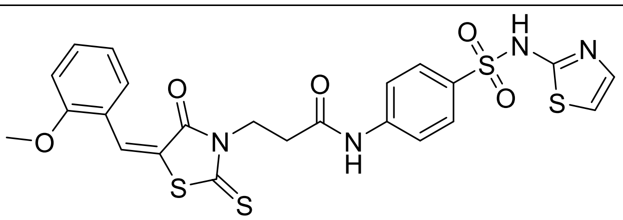	-41.191

Z18655491	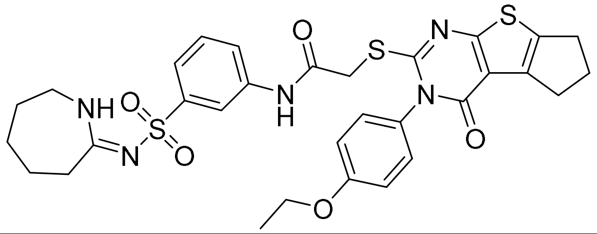	-41.0928
MolPort-004-946-102	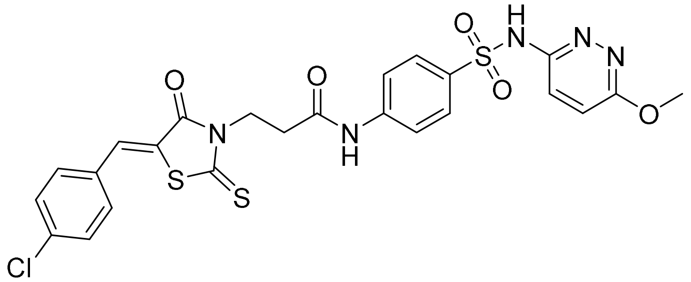	-41.0524
Amb1646445	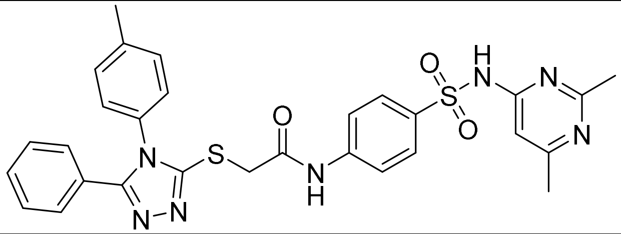	-40.8349
**5**<br>Amb3498025	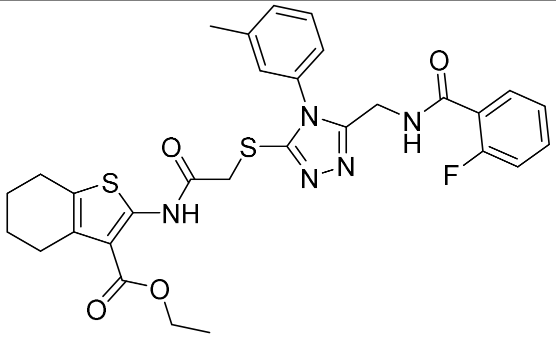	-40.6428
Z56990016	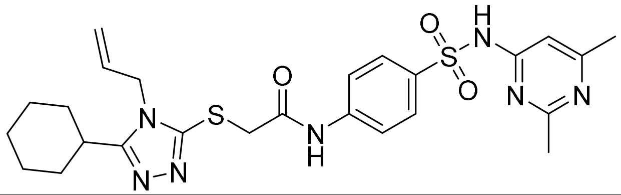	-40.5924
MolPort-001-570-727	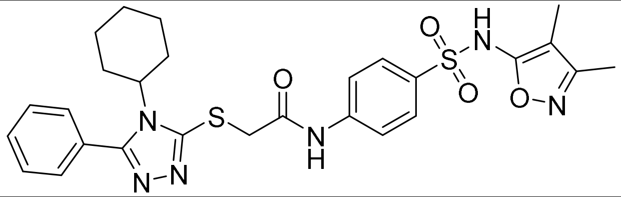	-40.5561
**6**<br>MolPort-000-805-473	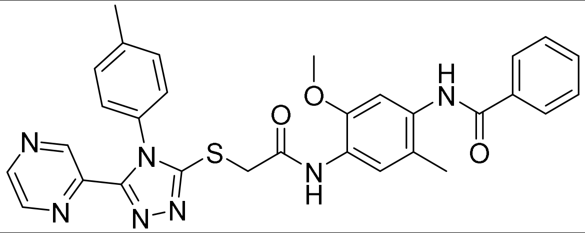	-40.4531

Amb3482451	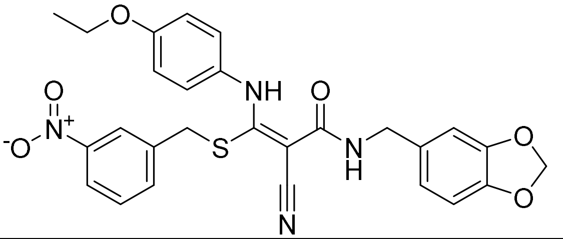	-40.4353
Amb416828	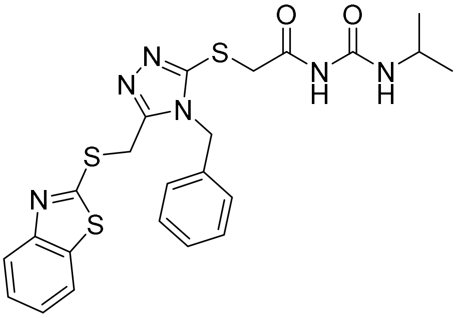	-40.3817
**7**<br>G613-0178	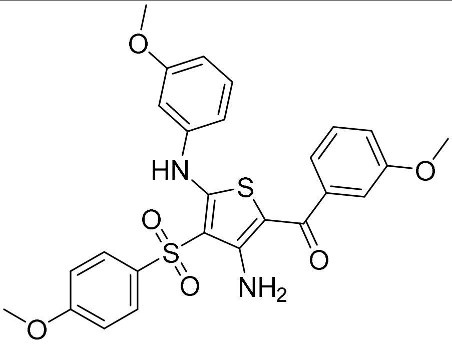	-40.3534
Amb30496753	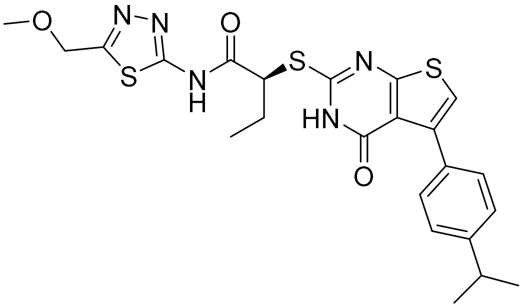	-40.205
MolPort-003-020-789	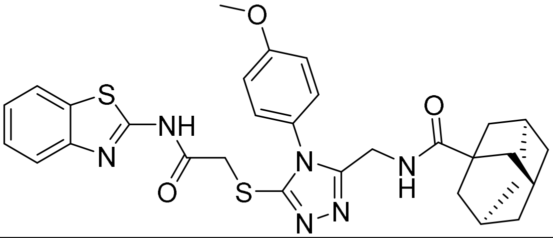	-40.1153
MolPort-000-779-437	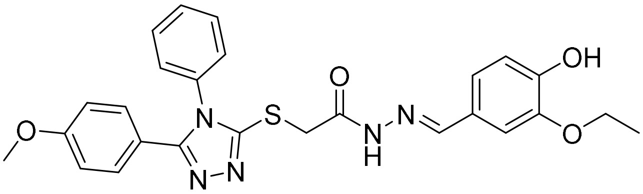	-40.0117

MolPort-000-440-480	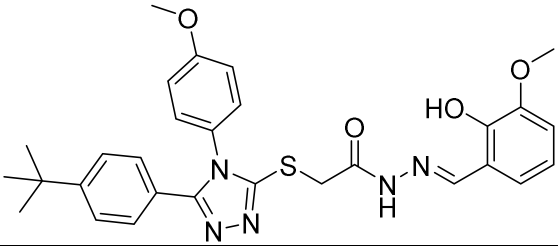	-39.8834
Amb21821692	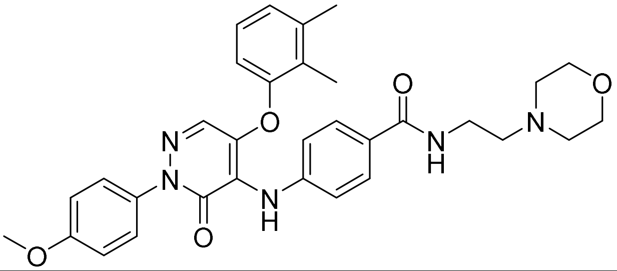	-39.8721
Z18622912	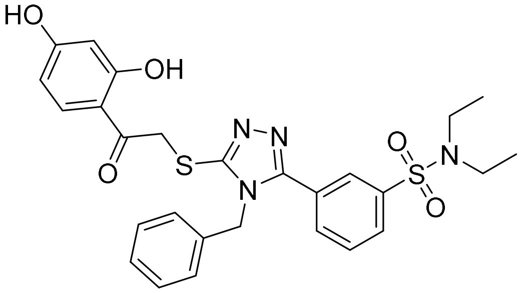	-39.8451
6208-0449	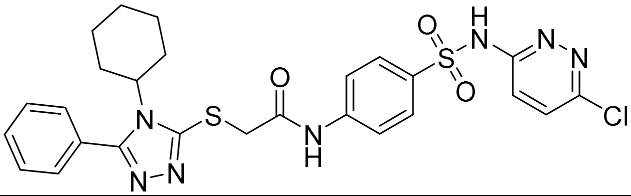	-39.7425
**8** MolPort-000-757-950	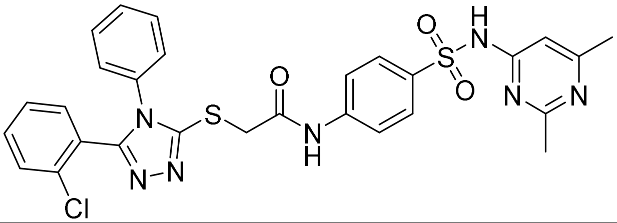	-39.6437
Amb31360914	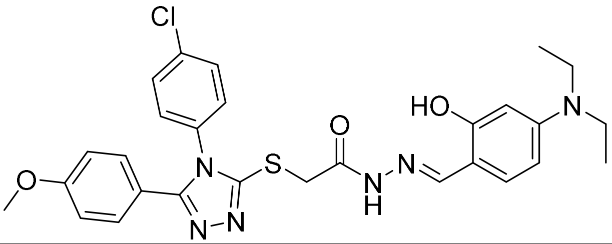	-39.4996
Z31293476	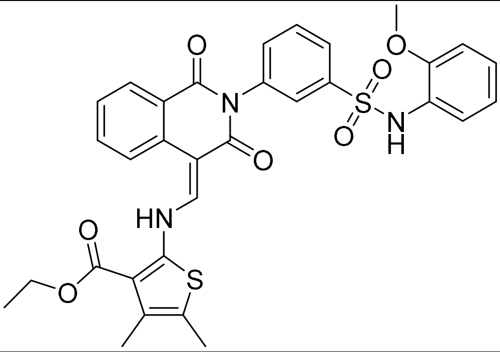	-39.4921

MolPort-006-536-405	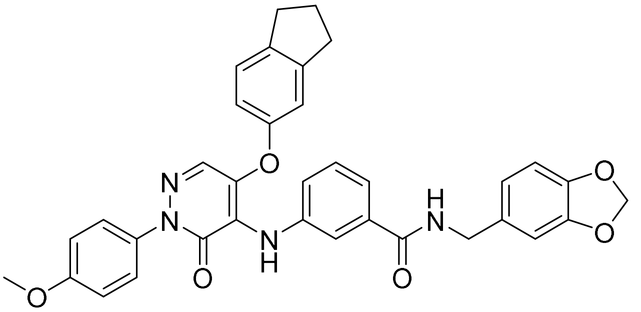	-39.2609
MolPort-000-439-418	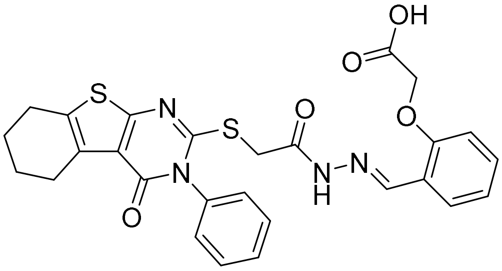	-39.2071
Amb31360799	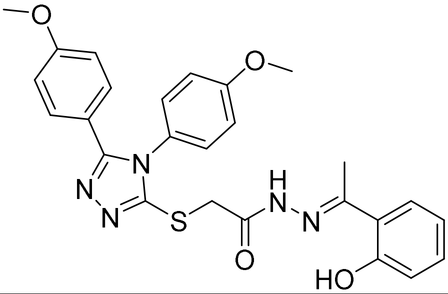	-39.1428
**9** V025-8452	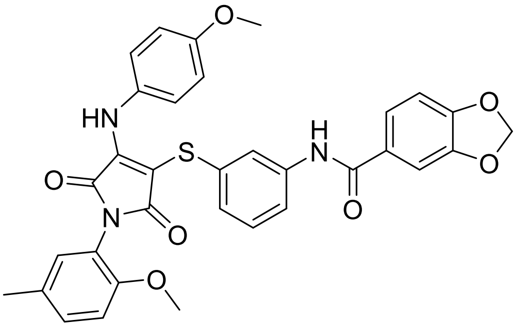	-38.9761
MolPort-047-898-743	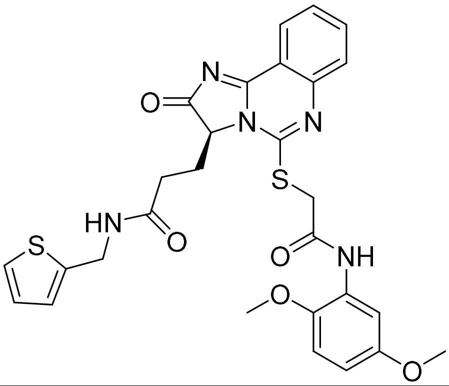	-38.9756

**10** MolPort-003-018-758	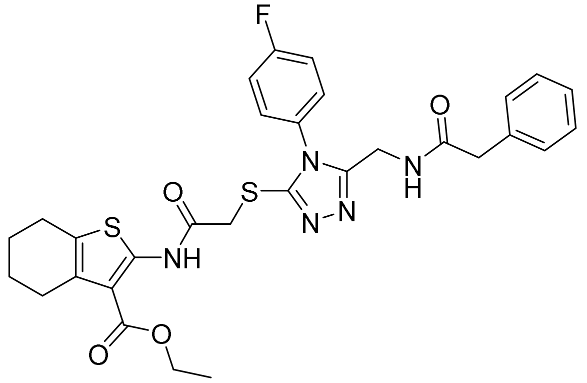	-38.8141
**11** Amb3422793	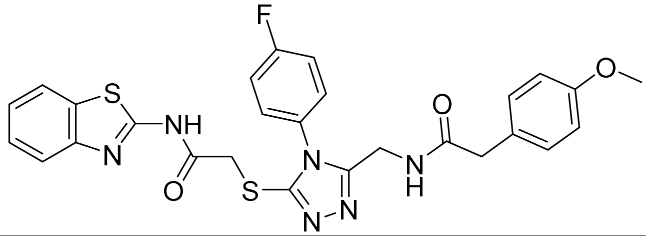	-38.8028
Amb31350053	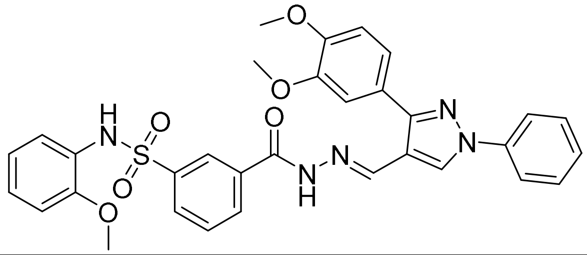	-38.7971
Amb23024289	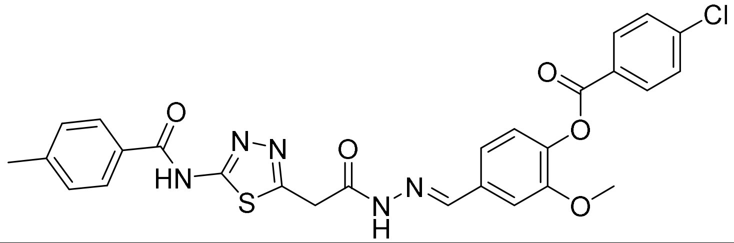	-38.6039

Table S5. The table shows the number and percentage of ligands out of a total of 40 from cluster 1 that exhibit a specific type of interaction with amino acid residues in the furin binding site.

AA residue (subsite)	H-bond		Pi-pi		Water mediated	
	number	%	number	%	number	%
Leu152	1	2.5				
Val231	5	12.5			5	12.5
Asp233 **(S6)**					15	37.5
Glu236 **(S4)**	15	37.5			8	20
Ser253	2	5				
Trp254	1	2.5	8	20		
Gly255					17	42.5
Pro256					4	10
Glu257 **(S5)**					13	32.5
Asp264 **(S4/S5)**	1	2.5			25	62.5
Gly265	2	5			9	22.5
Ala267	2	5			10	25
Trp291			7	17.5		

Tyr308	7	17.5				

Table S6. Top scoring compounds in cluster 2.

Cmpd	Structure	SCORE.INTER Score
**12** MolPort-003-054-063	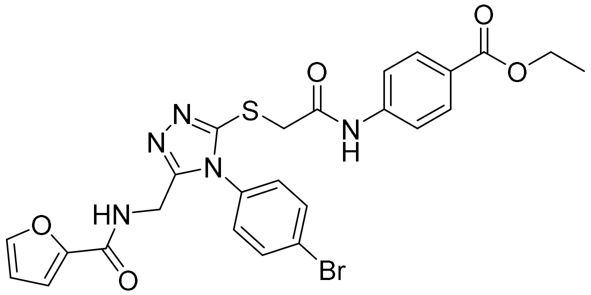	-46.3925
**13** MolPort-007-908-874	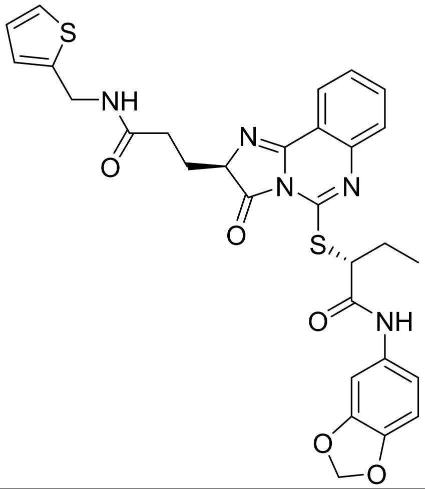	-42.0558
**14** Amb3476897	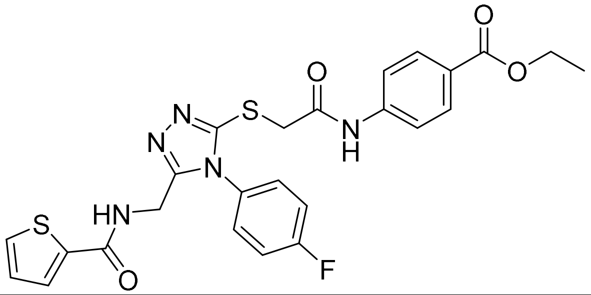	-41.4865
Amb2546270	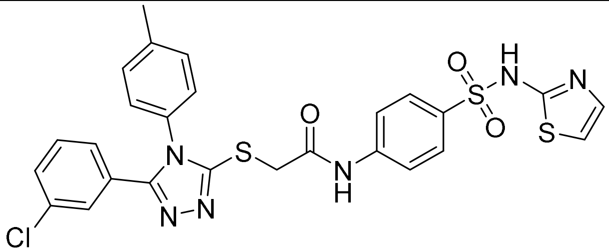	-41.1365

**15**<br>K250-0182	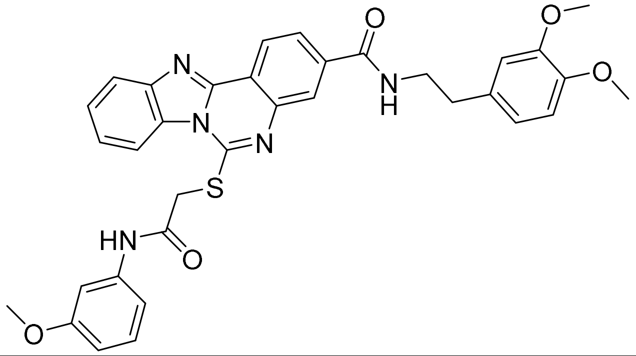	-40.1393
**16**<br>MolPort-000-802-159	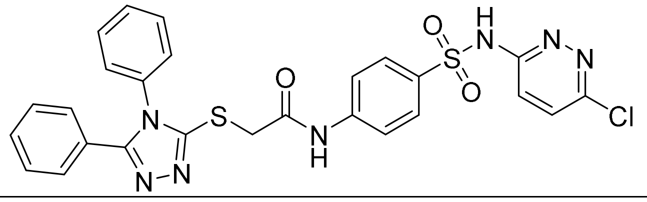	-40.079
**17**<br>MolPort-028-887-472	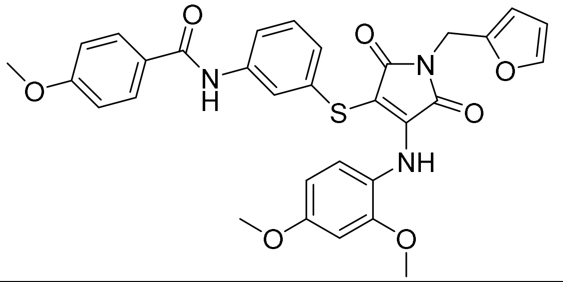	-39.7724
**18**<br>MolPort-001-969-537	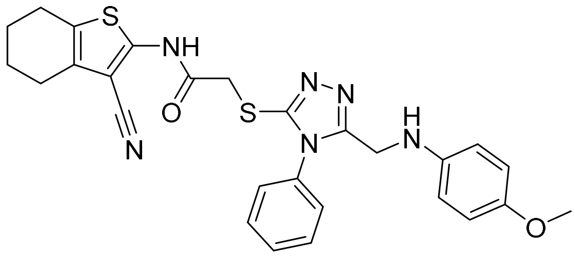	-39.4484
**19**<br>MolPort-003-052-514	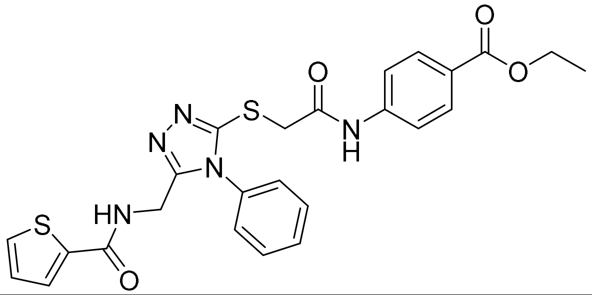	-39.4001

**20**<br>MolPort-003-071-523	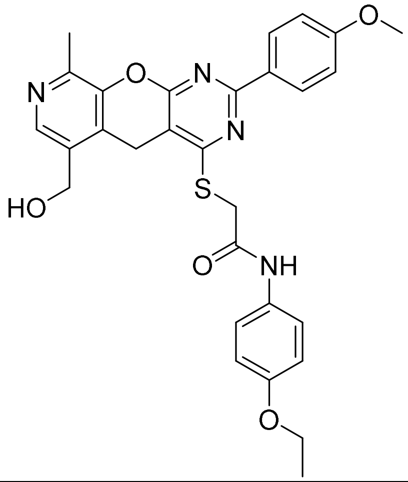	-38.6347

Table S7. The table shows the number and percentage of ligands out of a total of 10 from cluster 2 that exhibit a specific type of interaction with amino acid residues in the furin binding site.

AA residue (subsite)	H-bond		Pi-pi		Water mediated	
	number	%	number	%	number	%
Asp153					7	70
Asp154 **(S2)**					7	70
Asp191 **(S2)**					4	40
Arg193					1	10
Arg197					1	10
Val231	1	10			1	10
Asp233 **(S6)**					1	10
Glu236 **(S4)**	3	30				
Gly255					7	70
Pro256					2	20
Glu257 **(S5)**					3	30
Asp264 **(S4/S5)**			1	10	3	30
Tyr308	1	10	7	70		

Table S8. Percentage similarity of the top-scored compounds in clusters 1 and 2 with the known BOS inhibitors from Table S1.

**Cmpd**	**Morgan fingerprint similarity (%)**
MolPort-004-932-778	16.8
**1**<br>MolPort-003-017-240	13.5
**2**<br>Amb3424462	13.4
Amb395359	13
MolPort-002-946-465	16.2
Amb3424381	13
MolPort-028-893-466	13
MolPort-000-440-835	16.2
MolPort-000-445-638	13.9
**3**	15

Amb8607920	
**4**<br>MolPort-000-500-670	12.6
Z18655491	14.8
MolPort-004-946-102	13.4
Amb1646445	12.8
**5**<br>Amb3498025	12
Z56990016	15.2
MolPort-001-570-727	16.5
**6**<br>MolPort-000-805-473	15.7
Amb3482451	13.9
Amb416828	13.7
**7**<br>G613-0178	12.2
Amb30496753	12.1
MolPort-003-020-789	15.3
MolPort-000-779-437	15.5
MolPort-000-440-480	14.4
Amb21821692	15.8
Z18622912	13.8
6208-0449	15.7
**8**<br>MolPort-000-757-950	13.9
Amb31360914	15
Z31293476	12.6
MolPort-006-536-405	15.9
MolPort-000-439-418	14.9
Amb31360799	14.3
**9**<br>V025-8452	12.4
MolPort-047-898-743	14
**10**<br>MolPort-003-018-758	11.7
**11**<br>Amb3422793	14.1
Amb31350053	11.1
Amb23024289	12.6
**12**<br>MolPort-003-054-063	12.7
**13**<br>MolPort-007-908-874	13.3
**14**<br>Amb3476897	12.6
Amb2546270	15.8
**15**<br>K250-0182	10.5
**16**<br>MolPort-000-802-159	13.6

**17**<br>MolPort-028-887-472	13.2
**18**<br>MolPort-001-969-537	13
**19**<br>MolPort-003-052-514	12.9
**20**<br>MolPort-003-071-523	14.9

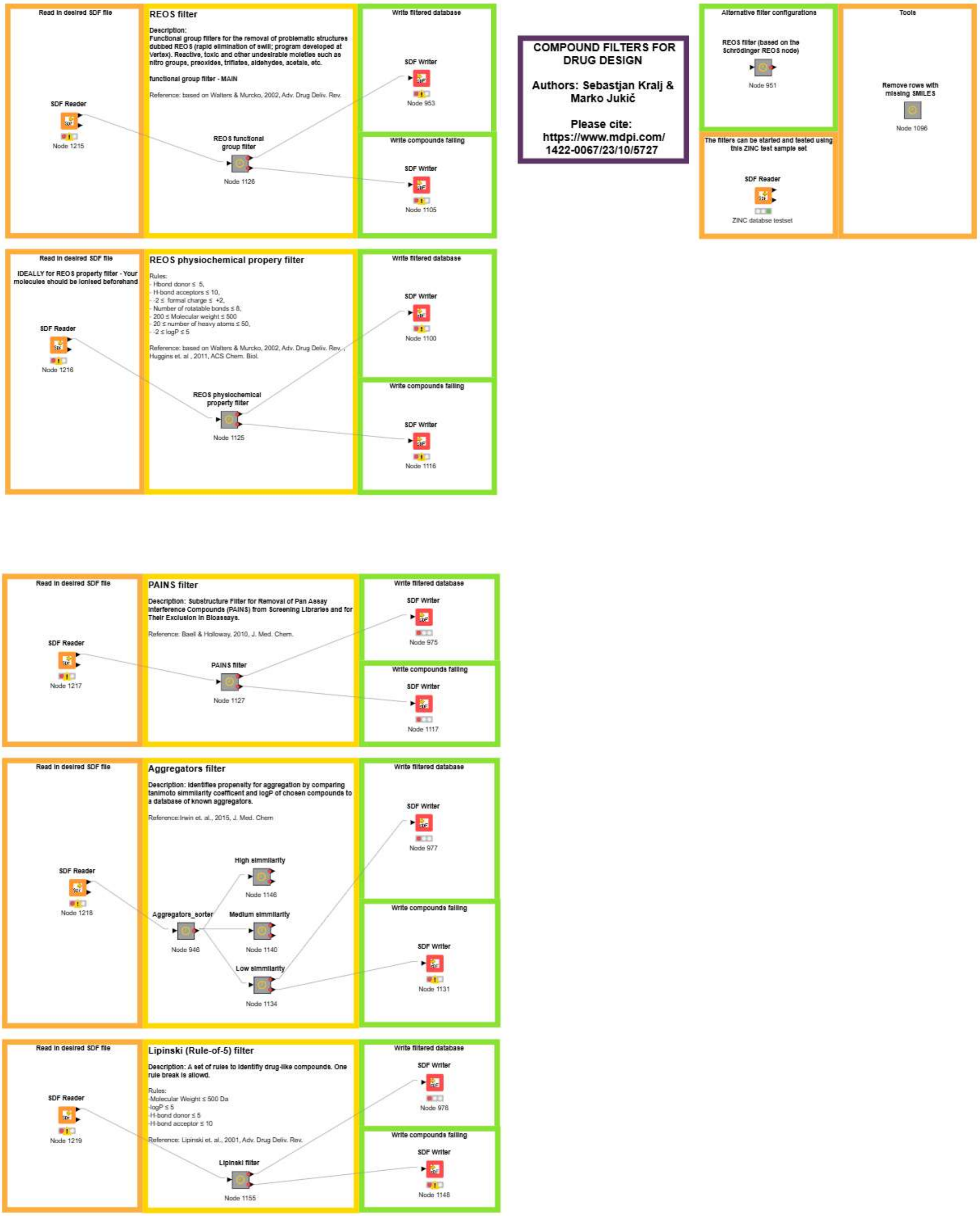

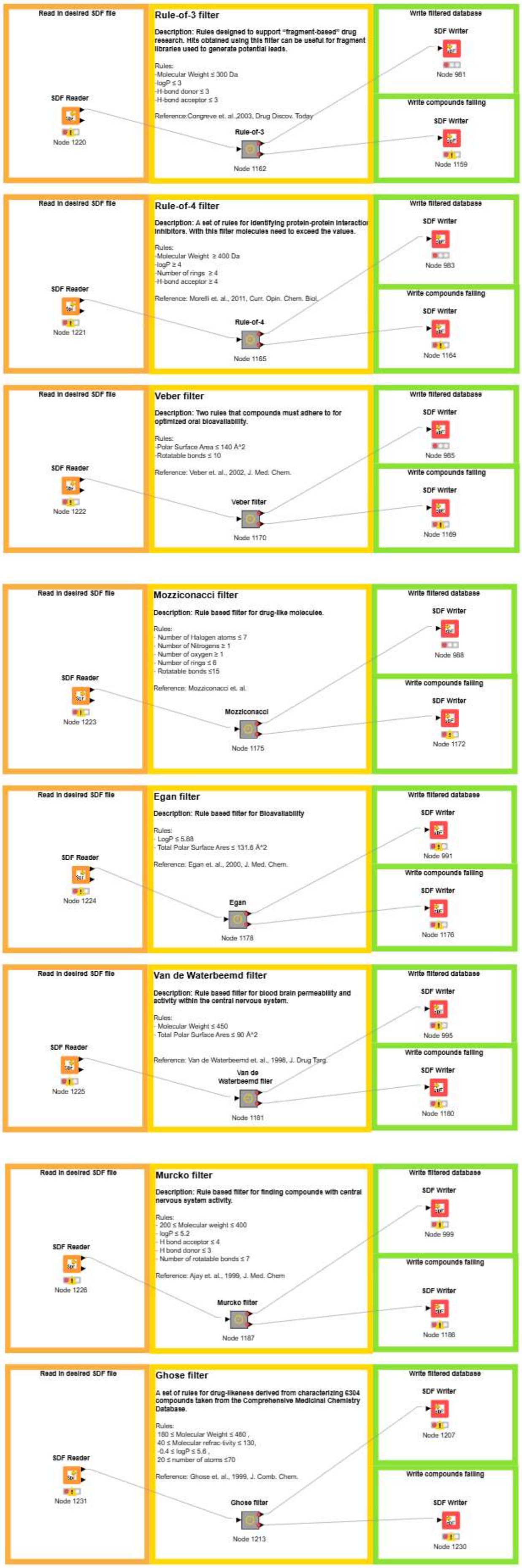
Read in desired SDF file
SDF Reader
Node 1220
Rule-of-3 filter
Description: Rules designed to support "fragment-based" drug research. Hits obtained using this filter can be useful for fragment libraries used to generate potential leads.
Rules:
-Molecular Weight ≤ 300 Da
-logP ≤ 3
-H-bond donor ≤ 3
-H-bond acceptor ≤ 3
Reference: Congreve et. al., 2003, Drug Discov. Today
Rule-of-3
Node 1162
Write filtered database
SDF Writer
Node 981
Write compounds failing
SDF Writer
Node 1159
Read in desired SDF file
SDF Reader
Node 1221
Rule-of-4 filter
Description: A set of rules for identifying protein-protein interaction inhibitors. With this filter molecules need to exceed the values.
Rules:
-Molecular Weight ≥ 400 Da
-logP ≥ 4
-Number of rings ≥ 4
-H-bond acceptor ≥ 4
Reference: Morelli et. al., 2011, Curr. Opin. Chem. Biol.
Rule-of-4
Node 1165
Write filtered database
SDF Writer
Node 983
Write compounds failing
SDF Writer
Node 1164
Read in desired SDF file
SDF Reader
Node 1222
Veber filter
Description: Two rules that compounds must adhere to for optimized oral bioavailability.
Rules:
-Polar Surface Area ≤ 140 A^2
-Rotatable bonds ≤ 10
Reference: Veber et. al., 2002, J. Med. Chem.
Veber filter
Node 1170
Write filtered database
SDF Writer
Node 985
Write compounds failing
SDF Writer
Node 1169
Read in desired SDF file
SDF Reader
Node 1223
Mozziconacci filter
Description: Rule based filter for drug-like molecules.
Rules:
- Number of Halogen atoms ≤ 7
- Number of Nitrogens ≥ 1
- Number of oxygen ≥ 1
- Number of rings ≤ 6
- Rotatable bonds ≤15
Reference: Mozziconacci et. al.
Mozziconacci
Node 1175
Write filtered database
SDF Writer
Node 988
Write compound failing
SDF Writer
Node 1172
Read in desired SDF file
SDF Reader
Node 1224
Egan filter
Description: Rule based filter for Bioavailability
Rules:
- LogP ≤ 5.88
- Total Polar Surface Area ≤ 131.6 A^2
Reference: Egan et. al., 2000, J. Med. Chem.
Egan
Node 1178
Write filtered database
SDF Writer
Node 991
Write compounds failing
SDF Writer
Node 1176
Read in desired SDF file
SDF Reader
Node 1225
Van de Waterbeemd filter
Description: Rule based filter for blood brain permeability and activity within the central nervous system.
Rules:
- Molecular Weight ≤ 450
- Total Polar Surface Area ≤ 90 A^2
Reference: Van de Waterbeemd et. al., 1998, J. Drug Targ.
Van de Waterbeemd filer
Node 1181
Write filtered database
SDF Writer
Node 995
Write compound failing
SDF Writer
Node 1180
Read in desired SDF file
SDF Reader
Node 1226
Murcko filter
Description: Rule based filter for finding compounds with central nervous system activity.
Rules:
- 200 ≤ Molecular weight ≤ 400
- logP ≤ 5.2
- H bond acceptor ≤ 4
- H bond donor ≤ 3
- Number of rotatable bonds ≤ 7
Reference: Ajay et. al., 1999, J. Med. Chem
Murcko filter
Node 1187
Write filtered database
SDF Writer
Node 999
Write compounds failing
SDF Writer
Node 1186
Read in desired SDF file
SDF Reader
Node 1231
Ghose filter
A set of rules for drug-likeness derived from characterizing 6304 compounds taken from the Comprehensive Medicinal Chemistry Database.
Rules:
180 ≤ Molecular Weight ≤ 480 ,
40 ≤ Molecular refrac-tivity ≤ 130,
-0.4 ≤ logP ≤ 5.6 ,
20 ≤ number of atoms ≤70
Reference: Ghose et. al., 1999, J. Comb. Chem.
Ghose filter
Node 1213
Write filtered database
SDF Writer
Node 1207
Write compounds failing
SDF Writer
Node 1230

Figure S1. KNIME workflow for agregator and REOS fitering.

The KNIME fitering workflow can be obtained at:
https://gitlab.com/Jukic/knime_medchem_filters [4].

Table S9. Re-docking validation experiment (3 top poses examined).

pdb_id	*lig_tricode*	*best_rmsd*	*best_score_rsmsd*	*scores*	*rmsds*	*lignad_index*
7QY0	I0T	0.8835836198620608	0.8835836198620608	[-38.8243, -38.7128, -37.602]	[0.8835836198620608, 1.174605967777508, 1.1319500621866037]	[3, 2, 1]
7QY1	I0W	0.4353283521536746	0.4353283521536746	[-45.9163, -44.5109, -41.0331]	[0.4353283521536746, 0.629222524996035, 1.5159501161689333]	[3, 2, 1]
7LCU	XTA	0.62912078678645	0.62912078678645	[-35.0454, -30.3694, -28.73]	[0.62912078678645, 0.7960498110205915, 2.120320091567643]	[3, 2, 1]
7QY2	I0Q	0.933044519507935	0.933044519507935	[-45.8485, -43.1768, -40.398]	[0.933044519507935, 1.6178481071648272, 1.2913603450272755]	[3, 2, 1]
7QXY	I1G	1.153059270554266	1.153059270554266	[-41.1509, -40.3565, -39.9318]	[1.153059270554266, 1.2581200230947902, 1.2855016303554743]	[3, 2, 1]
7QXZ	I0M	0.4096711885640089	1.3447022056292954	[-40.6888, -39.6937, -38.8574]	[1.3447022056292954, 0.47694009093548645, 0.4096711885640089]	[3, 2, 1]

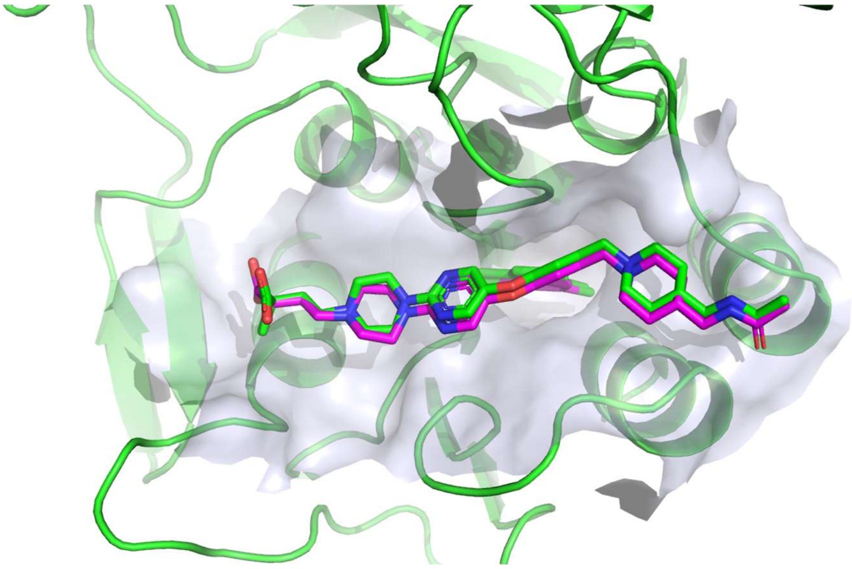

Figure S2. Re-docking of the 7QY2 I0Q small-molecule ligand with an RMSD of 0.93 Å. Re-docked ligand is represented in green stick model (with colored elements) while PDB ID: 7YQ2 crystal ligand conformation is presented in stick model colored magenta. Surrounding surface 6 Å around small-molecules is emphasized in transparent gray with protein secondary structure depicted in green colored model.

References

1. Osman, E.E.A.; Rehemtulla, A.; Neamati, N. Why all the fury over furin? J. Med. Chem. 2021, 65(4), 2747-2784.
2. Dahms, S.O.; Schnapp, G.; Winter, M.; Büttner, F.H.; Schlepütz, M.; Gnamm, C., Pautsch, A.; Brandstetter, H. Dichlorophenylpyridine-based molecules Inhibit furin through an induced-Fit mechanism. ACS Chem. Biol. 2022, 17(4), 816-821.
3. Axten, J.M.; Cheung, M.; Demartino, M.P.; Guan, H.A.; Hu, Y.; Miller, A.B.; Qin, D.; Wu, C., Zhang, Z., Lin, X. Furin inhibitors. WO2019215341A1, November 11, 2019.
4. Kralj, S., Jukič, M., & Bren, U. (2023). Molecular filters in medicinal chemistry. Encyclopedia, 3(2), 501-511.
